# Early postnatal changes in thyroid-stimulating hormone and subsequent neurodevelopment in preterm infants

**DOI:** 10.3389/fendo.2025.1745327

**Published:** 2026-02-03

**Authors:** Myoung-Jin Yoo, Yong Hun Jang, Gang-Yi Lee, Habyeong Kang, Dong Hye Ye, Woochang Hwang, Seung Yang, Hyun Ju Lee

**Affiliations:** 1 Department of Pediatrics, Hanyang University College of Medicine, Seoul, Republic of Korea; 2 Department of Pediatrics, Hanyang University Hospital, Seoul, Republic of Korea; 3 Department of Translational Medicine, Graduate School of Biomedical Science and Engineering, Hanyang University, Seoul, Republic of Korea; 4 Department of Preventive Medicine, College of Medicine, Hanyang University, Seoul, Republic of Korea; 5 Department of Computer Science, Georgia State University, Atlanta, GA, United States; 6 Department of Pre-Medicine, College of Medicine, Hanyang University, Seoul, Republic of Korea

**Keywords:** diffusion tensor imaging, neurodevelopmental impairment, preterm infants, thyroid function, TSH trajectory

## Abstract

**Introduction:**

Thyroid hormones are crucial for brain maturation during late gestation and early infancy. In preterm infants, immaturity of the hypothalamic–pituitary–thyroid axis often leads to transient or delayed dysfunctions undetected by standard newborn screening. As these atypical thyroid patterns have been associated with neurodevelopmental outcomes, serial assessments are warranted to better characterize risk. This study investigated whether thyroid trajectories between birth and discharge predict neurodevelopment at two years and their neural correlates on diffusion tensor imaging (DTI).

**Methods:**

This prospective cohort study included 222 preterm infants born at ≤32 weeks of gestation who underwent serial thyroid function tests at 1–2 weeks and at term-equivalent age or hospital discharge. Thyroid status was classified into quartile-based groups at each time point, and neurodevelopment at two years of corrected age was assessed using the BSID-III. Neurodevelopmental impairment (NDI) was defined as the presence of cerebral palsy, blindness, hearing loss, or a BSID-III cognitive or motor composite score below 85. Diffusion-tensor imaging at term-equivalent age was analyzed to examine brain network properties. Associations between longitudinal TSH quartile patterns and NDI were evaluated using logistic regression, with inverse probability of treatment weighting applied to adjust for baseline differences.

**Results:**

Infants with NDI had significantly higher thyroid-stimulating hormone (TSH) at birth and peak levels during hospitalization compared with typical development (*P* = 0.017 and *P* < 0.002). Cross-sectional analyses of TSH quartiles at newborn or TEA/discharge did not reveal independent associations with NDI after adjustment. In contrast, paired trajectories were more informative: infants with persistently low TSH, or those whose values declined from the interquartile to the lowest quartile by TEA/discharge, had a markedly lower risk of NDI (OR 0.24, *P* = 0.020; OR 0.23, *P* = 0.035). Brain DTI analyses suggested altered network centrality in the anterior cingulate and superior frontal gyri among infants with higher neonatal TSH.

**Conclusion:**

Preterm infants with persistently low or decreasing TSH trajectories showed reduced odds of neurodevelopmental impairment. In contrast, persistently high or increasing TSH levels may instead reflect alternative patterns of postnatal HPT-axis adaptation. Corresponding group differences in fronto-limbic regions at discharge provide neurobiological support that altered thyroid function may contribute to atypical brain connectivity underlying later neurodevelopmental outcomes.

## 1 Introduction

Thyroid hormones play a pivotal role in regulating overall growth and metabolism and are particularly essential for brain maturation during the third trimester and the first two years of life (1). Specifically, adequate thyroid hormone availability in the developing brain is critical during this period to support key neurodevelopmental processes such as neuronal migration, synaptogenesis, and myelination (2, 3). Disruption of the normal maturation of the hypothalamic–pituitary–thyroid (HPT) axis in preterm infants can therefore disturb hormonal homeostasis during this critical developmental window, potentially leading to enduring neurodevelopmental consequences.

Clinically, transient thyroid dysfunction in extremely (<28 weeks) and very preterm infants (28–32 weeks) has been associated with later neurodevelopmental delay (4–6). However, the evidence remains conflicting, largely reflecting methodological heterogeneity in when and how thyroid function is measured and the criteria applied to define abnormality across studies (6). For example, an analysis of 1,148 infants using newborn-screening data within 24–96 hours found that lower thyroid-stimulating hormone (TSH) quartiles predicted poorer motor outcomes (5). In contrast, a cohort study that evaluated thyroid status near discharge using gestational-age–adjusted TSH percentiles did not find consistent associations with cognitive or motor outcomes in BSID-III Scales of Infant and Toddler Development, Third Edition (BSID-III) (7). Despite these inconsistencies, neuroimaging studies have supported the hypothesis that thyroid imbalance may influence early brain connectivity, with altered thalamocortical connectivity observed in preterm infants with elevated TSH, suggesting subtle microstructural vulnerability linked to thyroid dysfunctions (8). Collectively, establishing reliable and accessible biomarkers that reflect thyroid trajectory and predict thyroid dysfunction–related neurodevelopmental impairment (NDI) is of substantial clinical importance.

Newborn screening (NBS) programs were implemented to detect congenital hypothyroidism (CH), which is characterized by insufficient thyroid hormone production at birth and can lead to metabolic and neurocognitive impairment, and to enable early levothyroxine replacement to prevent neurodevelopmental sequelae (9). However, in preterm infants, immaturity of the HPT axis, stress-related hormonal fluctuations, and the effects of critical illness can alter early thyroid responses, and as a result, standard screening programs often fail to detect the atypical, transient, or subclinical thyroid dysfunctions that differ from classic CH. These atypical thyroid phenotypes include delayed TSH elevation, in which TSH levels rise several weeks after birth despite initially normal FT4/T4 and T3; isolated hyperthyrotropinemia, defined by elevated TSH with normal FT4; and transient hypothyroxinemia of prematurity, defined by low FT4/T4 with normal or low TSH (6, 10, 11). Because these dynamic patterns often lead to false-negative results on initial screening, preterm infants are particularly prone to a delayed postnatal TSH rise that may not be detected even with a single follow-up sample. Consequently, recent endocrine consensus statements and population studies recommend serial thyroid screening in this population (9, 12–14). Supporting this recommendation, in a cohort with serial postnatal thyroid measurements at 7, 14, and 28 days and at 34 weeks’ corrected gestation, consistent mild thyroid dysfunction—elevated TSH or low thyroxine—was associated with 6–7 point reductions in BSID-III cognitive and motor scores at 2 years, a pattern not detected by standard newborn screening protocols (15).

Building upon these observations, the present study aimed to characterize thyroid function trajectories in preterm infants by integrating longitudinal assessments obtained at birth and at hospital discharge, and to examine their associations with the odds of NDI at two years of age. To strengthen the biological validity of these associations, we additionally incorporated diffusion tensor imaging (DTI)–derived structural connectome analyses to evaluate whether early thyroid alterations correspond to microstructural variations in brain connectivity. This combined clinical and imaging approach was designed to provide an integrated framework for understanding how early thyroid regulation patterns may influence later neurodevelopmental outcomes in preterm populations.

## 2 Methods

### 2.1 Study design and participants

This prospective cohort study included preterm infants born at ≤ 32 weeks of gestation who were admitted to the neonatal intensive care unit at Hanyang University Seoul Hospital between January 2016 and December 2024. A total of 243 infants were initially screened. Inclusion criteria were no intraventricular hemorrhage (grade 3-4), no chromosomal abnormalities, no congenital infections, and no cystic periventricular leukomalacia. After excluding 21 infants with poor-quality MRI data, 222 infants with complete paired thyroid and neuroimaging data were included in the final analysis. Serial thyroid function test (TFT) measuring both serum TSH and fT4 levels were routinely performed at 1~2 weeks of postnatal age and at term-equivalent age (TEA) or hospital discharge for all infants. Even if the initial TFT values obtained at 1~2 weeks of life were normal, we repeated the screening test at both 3~4 weeks and at TEA because delayed elevations of TSH elevation is common among premature infants. For each infant, the highest recorded TSH concentration during hospitalization was defined as the peak TSH. If the TFT values were abnormal (TSH>6 μU/mL and/or fT4<0.8 ng/dL), the test was repeated after 1 or 2 weeks according to careful consideration by the pediatric endocrinologists at our hospital. The Hanyang University Hospital Institutional Review Board (IRB No. 202101015) approved this study protocol, and informed consent for participation in this study was acquired by the parents. All procedures were performed in compliance with the principles of the Declaration of Helsinki.

### 2.2 Data collection and clinical variables

Maternal and neonatal characteristics were systematically recorded, including gestational age, postmenstrual age, birth weight, sex, mode of delivery, and maternal conditions such as gestational diabetes mellitus (GDM), pregnancy-induced hypertension (PIH), and chorioamnionitis. Maternal education was documented as a proxy for socioeconomic background. Clinical course variables and major morbidities during hospitalization were also captured, including hypoglycemia (blood glucose <40 mg/dL), respiratory distress syndrome (RDS), patent ductus arteriosus (PDA) and need for surgical ligation, intraventricular hemorrhage (IVH), periventricular leukomalacia (PVL), necrotizing enterocolitis (NEC) requiring surgery, bronchopulmonary dysplasia (BPD), and retinopathy of prematurity (ROP). Information regarding therapeutic exposures that may influence TSH levels, including levothyroxine therapy, dopamine therapy, and postnatal steroid use, was recorded. In our cohort, dopamine therapy was administered only to infants weighing <1,000 g who developed sepsis.

### 2.3 Thyroid function assessment

To evaluate relative thyroid status within the study population, infants were classified into quartile-based groups at each time point according to the distribution of TSH concentrations. At NBS, the quartile thresholds were defined as <2.97 μU/mL (lowest quartile), 2.97–6.92 μU/mL (interquartile range), and ≥6.92 μU/mL (highest quartile). At TEA or discharge, these thresholds were <2.37 μU/mL (lowest quartile), 2.37–4.76 μU/mL (interquartile range), and ≥ 4.76 μU/mL (highest quartile). This quartile-based classification was chosen instead of a fixed cutoff (e.g., TSH ≥ 6 μU/mL) to better represent the distributional variation and longitudinal trajectory of thyroid function across the cohort. Separately, CH was diagnosed according to institutional criteria (serum fT4 < 0.8 ng/dL and TSH > 20 μIU/mL) (16), based on results from newborn screening obtained at a mean postnatal age of 31 ± 33 days in our cohort. Among infants who required thyroid-related treatment, levothyroxine was initiated immediately after CH was confirmed.

### 2.4 Neurodevelopmental outcomes

At 24 months of corrected age, neurodevelopment was evaluated by neonatologists or pediatric rehabilitation physicians who were blinded to thyroid function data. The BSID-III was administered to assess cognitive, language, and motor domains. NDI was defined as the presence of at least one of the following: cerebral palsy diagnosed by abnormal tone with impaired gross motor function; significant hearing loss defined as a permanent hearing threshold greater than 55 dB in the better ear; blindness confirmed by ophthalmologic examination; or developmental delay defined as a BSID-III cognitive or motor composite score below 85 (5).

### 2.5 MRI acquisition

Preterm infants underwent MRI scanning at late preterm to near-term equivalent age (32–44 weeks PMA) using a 3T whole-body scanner (Achieva, Philips, Best, Netherlands) equipped with a 16-channel phased-array head coil. Imaging was performed during natural sleep, and infants were wrapped in a blanket to maintain body temperature. Throughout the procedure, a pediatrician monitored pulse oximetry to continuously assess respiratory and cardiac rates. For DTI, single-shot spin-echo three-dimensional echo-planar sequences were acquired. Acquisition parameters included: b-value = 1000 s/mm², echo time = 75 ms, repetition time = 4,800 ms, flip angle = 90°, field of view = 120 × 120 mm², voxel size = 1.56 × 1.56 mm², slice thickness = 2 mm, 16 diffusion directions distributed electrostatically, two signal averages, total scan time = 6 min 17 s, and water-fat shift = 4.68 Hz/pixel. Axial slices were aligned parallel to the anterior–posterior commissure line, with 40–50 slices covering the entire brain and brainstem. To quantify motion-related artifacts, diffusion-weighted datasets were evaluated using FSL’s EDDY QC tool (17) in the FMRIB software Library (FSL, https://fsl.fmrib.ox.ac.uk/fsl/fslwiki) (18), yielding indices such as absolute and relative framewise displacement and percentage of signal outliers.

### 2.6 Data preprocessing

Preprocessing of diffusion data involved correction for eddy current–related distortions, participant motion, and susceptibility-induced field inhomogeneities using an eddy correction tool. From the raw dataset, a nondiffusion-weighted (b0) volume was obtained, which still contained skull and extracerebral tissues. To correct for low-frequency intensity variations, the bias field was estimated and removed with the N4 bias field correction algorithm implemented in Advanced Normalization Tools (19). Diffusion tensor modeling was then performed by fitting the diffusion-weighted images with a simple least-squares approach, yielding the three eigenvalues of the tensor. These eigenvalues were subsequently used to compute standard diffusion metrics, including fractional anisotropy, mean diffusivity, axial diffusivity, and radial diffusivity. All preprocessed images underwent visual quality control by two independent raters.

### 2.7 Network construction

For spatial normalization, a 12-parameter affine registration was applied to align each subject’s b0 image with the T2-weighted template from the University of North Carolina (UNC) neonatal atlas (20). The reverse transformation was then used to warp the UNC automated anatomical labeling atlas into native space. To maintain discrete regional indices, nearest-neighbor interpolation—a sinc-based interpolation method—was employed. This procedure yielded 90 parcellated brain regions, each corresponding to a network node.

Whole-brain structural connectivity was reconstructed using probabilistic tractography implemented in FSL. First, BEDPOSTX was applied to model fiber orientations and account for partial volume effects related to slice thickness (parameters: three fibers per voxel; Rician noise model). Subsequently, PROBTRACKX was used to generate tractography on individual diffusion datasets. Connectivity matrices were constructed by computing the proportion of tracts originating from all voxels in region i and terminating in region j (PROBTRACKX parameters: 5,000 samples per seed voxel, step length = 0.5 mm, curvature threshold = 0.2, FA threshold = 0.01).

Because tractography outcomes are direction-dependent, the probability from region i to j does not necessarily equal that from j to i. To address this, we defined unidirectional connection probabilities (P_ij_) and averaged them to construct a symmetric 90 × 90 adjacency matrix. To minimize spurious low-probability links, pairwise Pearson correlations across participants were calculated for all 4,005 potential edges, and edges with r < 0.7 were excluded. Weighted edge strength (W_ij_) was finally defined as W_ij_ = P_ij_.

### 2.8 Global and local network analysis

Before computing graph-theoretical measures, a sparsity threshold of 0.25 was imposed on each subject’s network (21). This threshold, defined as the ratio of observed connections to the total number of possible edges, was chosen to suppress weak and potentially noise-driven links (22), following the procedure reported in our earlier work (23). Network characterization was then carried out using the Brain Connectivity Toolbox (24) in combination with the GRETNA package (http://www.nitrc.org/projects/gretna/) (25).

Both global and regional network features were assessed. We assessed the topological organization of neonatal structural connectomes using global and local graph measures. Specifically, global efficiency, local efficiency, clustering coefficient, shortest path length, and small worldness (24) were evaluated with respect to 1,000 random networks that preserved node count, edge count, and degree sequence under all sparsity thresholds (26). Local indices included betweenness centrality (BC) and degree centrality (DC) (27, 28). These metrics were considered sensitive markers of neonatal and pediatric brain development and were further analyzed to explore clinical significance (29–31). A more detailed description of these graph measures is provided in our previous publication (23).

### 2.9 Statistical analyses

Group-level demographic characteristics of preterm infants classified as typically developing (TD) or with NDI were initially compared using SPSS version 27.0 (SPSS Inc., Chicago, IL). To reduce potential baseline imbalances between groups, we further applied Inverse Probability of Treatment Weighting (IPTW) based on propensity scores derived from gestational age, postmenstrual age at scan, sex, maternal education, and free thyroxine levels at first. These covariates were selected *a priori* because they are known to influence both early thyroid function and neurodevelopment in preterm infants. Gestational maturity and baseline thyroid status affect HPT-axis development, maternal education reflects socioeconomic factors linked to developmental outcomes, and postmenstrual at MRI reduces variation related to maturation stage (6). Clinical variables were statistically evaluated using Student’s t-tests and chi-square tests both before and after IPTW adjustment, and the corresponding group-level statistics are reported.

Associations between TSH quartile patterns and NDI were evaluated using logistic regression models. The dependent variable was the presence or absence of NDI, and results were expressed as odds ratios with 95% confidence intervals and p-values. To address potential baseline imbalances, IPTW was applied using gestational age, postmenstrual age at scan, sex, maternal education, and first free thyroxine levels. Infants were categorized into nine subgroups according to the combination of TSH quartiles at newborn and TEA/discharge screening. The interquartile–interquartile group served as the reference, and odds of NDI were estimated for the remaining eight subgroups after balancing sample sizes through propensity score weighting. As a supplementary analysis, logistic regression was performed separately for TSH quartiles at newborn and at TEA/discharge, using the interquartile group as the reference. Odds ratios for NDI were reported before and after adjustment with IPTW.

For local network analysis, independent-samples t-tests were employed to evaluate differences between empirical and random networks. Comparisons of regional network topological measures across groups were conducted using one-way analysis of covariance (ANCOVA), with postmenstrual age at MRI included as a covariate. These analyses were implemented in R (R Core Team, https://www.r-project.org/).

## 3 Results

### 3.1 Clinical characteristics and neurodevelopmental outcomes

Infants with NDI had a significantly higher prevalence of PDA ligation compared with those with TD before IPTW adjustment (78/95 [82.7%] *vs*. 66/127 [69.5%], P = 0.021, **Table 1**). After weighting by gestational age, sex, postmenstrual age at MRI, maternal education, and newborn fT4 levels (IPTW adjustment), dopamine therapy differed significantly between groups (7.2% *vs*. 2.3%, P = 0.024), and despite this higher likelihood of hemodynamic compromise, the group with greater dopamine exposure demonstrated neurodevelopmental outcomes consistent with the TD group, suggesting that hypotension-related hypoperfusion is unlikely to fully explain the observed associations. Postnatal steroid exposure remained similar between groups. No significant between-group differences were found in other maternal or neonatal characteristics before or after IPTW, including gestational age, birth weight, sex, mode of delivery, or major morbidities such as hypoglycemia, RDS, BPD, IVH, NEC requiring surgery, or ROP, indicating that these perinatal factors were well balanced between groups and unlikely to confound the observed associations. For CH, 23 subjects (10.3%) met diagnostic criteria, and all received levothyroxine, typically initiated at 10–15 μg/kg/day. Since treatment was given exclusively to infants with CH, the distribution of levothyroxine exposure followed that of CH. CH prevalence did not differ between TD and NDI groups before or after IPTW, and levothyroxine exposure showed the same pattern.

**Table 1:** Clinical characteristics of preterm infants.

Variables	Unweighted study population			Weighted study population		
	TD (N = 127)	NDI (N = 95)	*P-value*	TD (N = 127)	NDI (N = 95)	*P-value*
Gestational age, weeks	28.15 ± 2.47	27.94 ± 2.62	0.564	27.82 ± 2.28	27.84 ± 2.70	0.954
Postmenstrual age, weeks	37.53 ± 2.74	37.65 ± 2.56	0.754	37.76 ± 2.48	37.70 ± 2.13	0.878
Birth weight, g	1171.21 ± 343.26	1194.41 ± 441.95	0.680	1120.19 ± 288.49	1098.18 ± 301.85	0.666
Male sex, n (%)	59 (43.1%)	39 (45.9%)	0.786	53.6 (39.9%)	55.7 (40.5%)	0.929
Cesarean section, n (%)	114 (83.2%)	70 (82.4%)	1.000	102.3 (76.2%)	115.5 (83.8%)	0.111
Single, n (%)	92 (67.2%)	55 (64.7%)	0.920	92.1 (68.5%)	90.1 (65.5%)	0.804
Dopamine therapy, n (%)	8 (6.3%)	3 (3.2%)	0.404	15.4 (7.2%)	5.1 (2.3%)	0.024*
Postnatal steroid, n (%)	88 (64.2%)	52 (61.2%)	0.752	87 (64.7%)	81 (58.8%)	0.315
Levothyroxine treatment, n (%)	14 (11.0%)	9 (9.5%)	0.810	25.0 (35.5%)	17.5 (41.0%)	0.090
Hypoglycemia, n (%)	8 (6.3%)	5 (5.3%)	0.810	7.9 (6.1%)	4.9 (5.3%)	0.697
Congenital hypothyroidism, n (%)	14 (11.0%)	9 (9.5%)	0.810	25.0 (35.5%)	17.5 (41.0%)	0.090
RDS, n (%)	105 (83.2%)	79 (82.6%)	1.000	179.5 (83.7%)	179.5 (82.5%)	0.731
PDA, n (%)	99 (72.3%)	55 (64.7%)	0.299	99.5 (74.1%)	88.3 (64.2%)	0.077
PDA ligation, n (%)	66 (69.5%)	78 (82.7%)	0.021*	22.8 (18.1%)	24.9 (26.2%)	0.148
GDM, n (%)	20 (14.6%)	11 (12.9%)	0.883	14.7 (10.9%)	7.1 (5.2%)	0.080
PIH, n (%)	25 (18.2%)	9 (10.6%)	0.177	18.3 (13.6%)	18.1 (13.2%)	0.910
Chorioamnionitis, n (%)	42 (30.7%)	21 (24.7%)	0.422	55.7 (41.5%)	44 (31.9%)	0.102
BPD moderate, n (%)	34 (26.8%)	34 (35.8%)	0.961	36.3 (28.8%)	31.0 (32.5%)	0.546
IVH, n (%)	35 (27.6%)	31 (32.6%)	0.413	37.5 (29.8%)	29.4 (30.8%)	0.860
NEC operation, n (%)	4 (3.1%)	4 (4.2%)	0.727	4.7 (3.7%)	3.2 (3.4%)	0.909
ROP, n (%)	26 (20.5%)	16 (16.8%)	0.494	25.5 (20.2%)	15.3 (16.0%)	0.426
BSID-III						
Cognition	98.11 ± 13.43	97.59 ± 14.00	0.809	98.72 ± 13.41	97.74 ± 14.58	0.696
Language	92.03 ± 12.65	90.86 ± 13.45	0.574	91.55 ± 12.34	90.34 ± 12.73	0.586
Motor	100.78 ± 13.59	97.58 ± 16.51	0.189	100.24 ± 13.49	97.60 ± 17.19	0.337
Maternal education, n (%)			0.535			0.739
> 6 years	1 (0.8%)	1 (1.1%)		1.1 (0.8%)	0.7 (0.7%)	
< 12 years	0 (0%)	0 (0%)		0 (0%)	0 (0%)	
< 16 years	68 (53.5%)	43 (45.3%)		10.4 (8.2%)	13.2 (13.8%)	
> 16 years	47 (37.0%)	38 (40.0%)		68.3 (54.1%)	42.8 (44.7%)	
at the early-postnatal (1–2 weeks) TSH, μIU/mL	4.79 ± 2.742	5.98 ± 4.087	0.017*	4.96 ± 3.09	5.61 ± 3.73	0.058
Discharge TSH, μIU/ml	3.67 ± 2.09	4.00 ± 2.34	0.235	3.60 ± 2.29	4.09 ± 2.33	0.246
Peak TSH, μIU/ml	5.62 ± 2.97	7.53 ± 4.83	<0.002*	5.74 ± 3.42	6.89 ± 4.37	<0.002*
at the early-postnatal (1–2 weeks) fT4, ng/dL	1.28 ± 0.37	1.35 ± 0.50	0.230	1.27 ± 0.33	1.29 ± 0.49	0.914
Discharge fT4, ng/dL	1.37 ± 0.24	1.39 ± 0.23	0.641	0.35 ± 0.24	1.35 ± 0.25	0.957

Data are presented as the mean ± SD or number (%). IPTW was applied based on propensity scores derived from gestational age, postmenstrual age at MRI, sex, maternal education, and free thyroxine levels at the first postnatal thyroid function test. Abbreviations: BSID-III, Bayley Scales of Infant and Toddler Development, Third Edition; BPD, bronchopulmonary dysplasia; fT4, free thyroxine; GDM, gestational diabetes mellitus; IVH, intraventricular hemorrhage; NDI, neurodevelopmental impairment; NEC, necrotizing enterocolitis; RDS, respiratory distress syndrome; PDA, patent ductus arteriosus; PIH, pregnancy-induced hypertension; PVL, periventricular leukomalacia; TD, typical development; TSH, thyroid-stimulating hormone; SD, standard deviation; IPTW, inverse probability of treatment weighting

The symbol (*) indicates statistical significance at p < 0.05.

With regard to thyroid function, mean TSH at NBS was higher in the NDI group compared with TD (5.98 ± 4.09 *vs*. 4.79 ± 2.74 μU/mL, *P* = 0.017). However, after applying IPTW, this difference did not reach significance (*P* = 0.058). TSH concentrations at TEA/discharge were not significantly different between the groups. In contrast, peak TSH remained consistently higher in the NDI group both unweighted (7.53 ± 4.83 *vs*. 5.62 ± 2.97, *P* < 0.002) and weighted (6.89 ± 4.37 *vs*. 5.74 ± 3.42, *P* < 0.002) analyses, suggesting that peak TSH during hospitalization may carry stronger prognostic factor of NDI.

### 3.2 NDI risks by TSH quartiles

When infants were stratified by quartiles at NBS, those in the lowest quartile (<2.97 μU/mL, n = 56) showed a slightly higher proportion of NDI compared with the interquartile group (41% *vs*. 38%; OR 1.13, *P* = 0.718; adjusted *P* = 0.657) (**Supplementary Table 1**). Infants in the highest quartile (≥6.92 μU/mL, n = 56) demonstrated a higher proportion of NDI (53% *vs*. 38%; OR 1.87, *P* = 0.060), but this difference was not statistically significant after adjustment (*P* = 0.303). At TEA/discharge, neither the lowest quartile (OR 0.68, *P* = 0.256) nor the highest quartile (OR 1.03, *P* = 0.935) differed significantly from the interquartile range. IPTW-adjusted results confirmed these findings, showing no significant differences across quartiles at either time point.

Following IPTW adjustment, thyroid function remained the only variable showing significant group differences. The NDI group exhibited higher TSH concentrations at both NBS (5.61 ± 3.73 *vs*. 4.96 ± 3.09 μU/mL) and peak TSH during hospitalization (6.89 ± 4.37 *vs*. 5.74 ± 3.42 μU/mL), whereas fT4 levels did not differ significantly between groups.

### 3.3 NDI risks by paired TSH trajectories

Longitudinal analysis of paired TSH quartiles revealed statistically significant trends associated with NDI (**Table 2**). Infants who remained in the lowest quartile across both time points (propensity score matched n = 24) had a markedly reduced risk of NDI (25.0%; OR 0.24, *P* = 0.020) compared with the interquartile across both time points. A similar protective effect was observed in those whose TSH shifted downward from the interquartile range at NBS to the lowest quartile by TEA/discharge (propensity score matched n = 20, 35.0%; OR 0.23, *P* = 0.035). These findings indicate that persistently low or declining TSH trajectories are associated with a lower likelihood of NDI.

**Table 2:** Risk of neurodevelopmental impairment by newborn and TEA/discharge TSH quartiles.

TSH/discharge screening	Matched Infants, N	Neurodevelopmental impairment, N (%)	Odds ratio (CI)	*P*-value
Newborn screening group: Lowest quartile (TSH < 2.97 μU/mL)				
TEA/discharge screening group				
Lowest quartile	24	6 (25.0%)	0.24 (0.07-0.80)	0.020*
Interquartile	21	11 (61.1%)	0.68 (0.19-2.36)	0.541
Highest quartile	5	3 (60.0%)	NA	NA
Newborn screening group: Interquartile (2.97 ≤ TSH < 6.92 μU/mL)				
TEA/discharge screening group				
Lowest quartile	20	7 (35.0%)	0.23 (0.06-0.90)	0.035*
Interquartile	61	26 (40.6%)	Reference	
Highest quartile	24	6 (28.6%)	0.42 (0.12-1.46)	0.174
Newborn screening group: Highest quartile (TSH ≥ 6.92 μU/mL)				
TEA/discharge screening group				
Lowest quartile	7	4 (57.1%)	NA	NA
Interquartile	19	8 (53.3%)	0.51 (0.16-1.67)	0.268
Highest quartile	22	11 (55.0%)	0.83 (0.24-2.89)	0.771

NA, In subgroups with extremely small samples, 95% confidence intervals and p-values could not be obtained (N/A) and were excluded from the report. Abbreviations: TEA, term-equivalent age; TSH, thyroid-stimulating hormone; CI, confidence interval; NA, not available.

The symbol (*) indicates statistical significance at p < 0.05.

In contrast, neurodevelopmental outcomes did not differ significantly among infants with higher or fluctuating TSH trajectories. Infants who started in the highest quartile but normalized to the interquartile range at TEA/discharge (propensity score matched n = 19) showed a comparable rate of NDI (53.3%; OR 0.51, *P* = 0.268), and those with persistently high TSH levels demonstrated no meaningful difference in NDI risk (55.0%; OR 0.83, *P* = 0.771).

### 3.4 Brain network analysis

Brain network analysis revealed regional differences between infants stratified by NBS TSH quartiles at birth (**Table 3**). In measures of BC, the highest TSH quartile group showed higher values in the right anterior cingulate gyrus (ACG) (48.080 ± 36.225 *vs*. 38.050 ± 27.734, *P* = 0.027) and lower values the left superior medial frontal gyrus (SFGmed) (88.063 ± 50.834 *vs*. 110.877 ± 64.962, *P* = 0.016) compared with the lower-quartile group. In DC analyses, the highest-quartile group exhibited higher values in the right ACG (32.375 ± 6.741 *vs*. 29.735 ± 7.412, *P* = 0.014) and lower values in the right middle frontal gyrus (MFG) (22.717 ± 7.595 *vs*. 25.036 ± 6.461, *P* = 0.024). No significant differences were observed in global network measures between groups (**Supplementary Table 2**).

**Table 3:** Regional differences in local brain network by newborn screening TSH quartile groups.

Metric	Region	Lowest to interquartile (N = 166)	Highest quartile (N = 56)	*P*-value
BC	Anterior cingulate gyrus, left	56.490 ± 44.180	57.573 ± 44.003	0.686
	Anterior cingulate gyrus, right	38.050 ± 27.734	48.080 ± 36.225	0.027*
	Amygdala, left	40.071 ± 27.654	45.679 ± 27.777	0.132
	Amygdala, right	49.039 ± 30.000	45.853 ± 23.755	0.568
	Hippocampus, left	41.853 ± 35.296	41.745 ± 41.565	0.905
	Hippocampus, right	21.318 ± 15.133	22.982 ± 18.339	0.508
	Inferior frontal gyrus (opercular), left	28.644 ± 23.374	26.164 ± 23.178	0.477
	Inferior frontal gyrus (opercular), right	11.131 ± 11.616	9.987 ± 8.037	0.401
	Inferior frontal gyrus (triangular), left	68.707 ± 63.271	56.054 ± 43.145	0.174
	Inferior frontal gyrus (triangular), right	93.196 ± 56.439	90.474 ± 51.491	0.717
	Insula, left	40.290 ± 43.943	40.619 ± 47.595	0.757
	Insula, right	22.804 ± 23.572	26.527 ± 22.757	0.208
	Middle frontal gyrus, left	29.010 ± 34.356	27.552 ± 34.102	0.876
	Middle frontal gyrus, right	17.908 ± 22.687	24.277 ± 26.060	0.078
	Superior frontal gyrus (dorsal), left	10.967 ± 10.796	9.267 ± 8.372	0.288
	Superior frontal gyrus (dorsal), right	14.218 ± 14.439	13.602 ± 10.933	0.665
	Superior frontal gyrus (medial), left	110.877 ± 64.962	88.063 ± 50.834	0.016
	Superior frontal gyrus (medial), right	72.110 ± 41.256	79.663 ± 49.154	0.293
	Orbitofrontal cortex (middle), left	88.700 ± 43.507	84.286 ± 41.829	0.410
	Orbitofrontal cortex (middle), right	67.788 ± 46.473	75.243 ± 38.605	0.341
	Orbitofrontal cortex (medial), left	2.689 ± 5.771	3.211 ± 6.405	0.505
	Orbitofrontal cortex (medial), right	3.016 ± 5.660	4.558 ± 11.190	0.168
	Superior temporal gyrus, left	21.365 ± 24.701	20.285 ± 23.401	0.969
	Superior temporal gyrus, right	55.033 ± 53.006	56.152 ± 59.663	0.671
DC	Anterior cingulate gyrus, left	32.024 ± 7.437	32.821 ± 8.295	0.396
	Anterior cingulate gyrus, right	29.735 ± 7.412	32.375 ± 6.741	0.014*
	Amygdala, left	23.614 ± 8.005	24.571 ± 8.139	0.348
	Amygdala, right	25.687 ± 5.866	25.589 ± 6.483	0.994
	Hippocampus, left	29.277 ± 7.357	29.411 ± 6.983	0.873
	Hippocampus, right	24.982 ± 5.574	26.036 ± 5.982	0.203
	Inferior frontal gyrus (opercular), left	26.608 ± 6.220	26.000 ± 6.161	0.461
	Inferior frontal gyrus (opercular), right	21.434 ± 5.383	21.679 ± 4.260	0.968
	Inferior frontal gyrus (triangular), left	32.958 ± 7.897	32.536 ± 6.814	0.713
	Inferior frontal gyrus (triangular), right	37.861 ± 7.073	38.250 ± 7.043	0.649
	Insula, left	28.072 ± 7.862	27.946 ± 8.394	0.899
	Insula, right	25.542 ± 7.596	27.250 ± 7.948	0.085
	Middle frontal gyrus, left	25.518 ± 7.373	25.286 ± 8.022	0.824
	Middle frontal gyrus, right	25.036 ± 6.461	22.717 ± 6.595	0.024*
	Superior frontal gyrus (dorsal), left	22.783 ± 6.532	23.179 ± 5.454	0.601
	Superior frontal gyrus (dorsal), right	24.657 ± 6.481	25.625 ± 6.068	0.301
	Superior frontal gyrus (medial), left	39.922 ± 7.670	38.571 ± 6.578	0.33
	Superior frontal gyrus (medial), right	35.404 ± 6.394	36.643 ± 7.482	0.231
	Orbitofrontal cortex (middle), left	34.054 ± 8.279	33.786 ± 7.769	0.838
	Orbitofrontal cortex (middle), right	31.283 ± 7.295	32.982 ± 7.379	0.164
	Orbitofrontal cortex (medial), left	13.343 ± 5.328	13.518 ± 5.705	0.820
	Orbitofrontal cortex (medial), right	13.572 ± 5.924	14.518 ± 7.209	0.265
	Superior temporal gyrus, left	24.277 ± 8.031	25.000 ± 7.206	0.444
	Superior temporal gyrus, right	30.663 ± 9.309	30.661 ± 10.195	0.829

TSH, thyroid-stimulating hormone; BC, betweenness centrality; DC, degree centrality.

The symbol (*) indicates statistical significance at p < 0.05.

## 4 Discussion

In this study, we investigated the longitudinal patterns of thyroid function in preterm infants by comparing TSH levels at birth and at discharge, and examined their associations with neurodevelopmental outcomes and brain connectivity. Infants with persistently low or mid-to-low TSH trajectories demonstrated lower odds of neurodevelopmental impairment at 24 months, whereas those with elevated or nondeclining TSH patterns exhibited higher risk. Notably, infants with higher peak TSH values showed alterations in frontal–cingulate network centrality on diffusion-tensor imaging, providing neurobiological evidence that early thyroid dysregulation may influence cortical network maturation.

In our cohort, infants with NDI exhibited distinctly higher peak TSH concentrations, in most cases, compared with those with typical development. Notably, several cohort and neuroimaging studies have demonstrated that even modest elevations in neonatal TSH, particularly peaks exceeding 6 μU/mL, are associated with later neurodevelopmental or structural brain alterations. In large population-based analyses, infants with borderline-high TSH values below conventional screening thresholds showed lower cognitive and educational performance at school age (32), while other prospective cohorts reported associations between higher neonatal TSH and poorer developmental scores during infancy (33). Neuroimaging data further link such elevations to disrupted thalamocortical and frontal–cingulate connectivity in preterm infants, supporting a mechanistic bridge between thyroid immaturity and altered cortical network development (8). This pattern aligns with mechanistic insights described in recent reviews, which emphasize that even mild disturbances of the thyroid axis in preterm infants may influence key neurodevelopmental processes, including glial maturation, myelination, and cortical organization (34). Specifically, thyroid hormones are essential for early cortical development, and T3 has been shown to regulate Cajal–Retzius and subplate cell–mediated cortical layering, axonal guidance, and neuronal migration (3). Moreover, TH signaling supports oligodendrocyte and astrocyte maturation and promotes myelination and synaptogenesis during the late gestational period (35, 36). Given that preterm birth interrupts these critical windows, even modest deviations in neonatal thyroid function may exert disproportionate effects on early brain network formation. Collectively, these findings suggest that a peak TSH elevation represents a biologically sensitive threshold of HPT axis immaturity with measurable consequences for brain organization and later neurodevelopment.

Given the established association between higher peak TSH levels and later neurodevelopmental vulnerability, we next examined whether longitudinal changes in TSH between newborn and term-equivalent age could further refine the prediction of neurodevelopmental outcomes. Our present results exhibited that repeated assessment of TSH between newborn and TEA in our cohort provided additional prognostic information that a single measurement could not. Notably, the present study exhibited that infants who remained in the lower-to-mid interquartile or showed a gradual decline in TSH across these two time points had lower odds of NDI. Furthermore, across outcome studies, persistently abnormal or rising TSH across serial assessments has been linked to lower BSID-III scores at 2 years, whereas stable low-to-mid trajectories have not shown excess risk; however, among extremely preterm infants, persistently lowest-quartile TSH at birth and discharge has been associated with higher NDI risk (5, 15). This longitudinal pattern suggests that early postnatal stabilization of the HPT axis—reflecting efficient feedback adaptation after the initial TSH and T4 surge (6, 37)—may be critical for supporting subsequent neurodevelopment (38, 39). This interpretation aligns with established endocrine physiology describing a transient rise in TSH and T4 immediately after birth, followed by a gradual attainment of homeostatic equilibrium over the ensuing weeks. In contrast, infants whose TSH levels remained elevated or continued after an initial rise did not show a comparable advantage. This trajectory indicates incomplete maturation or stress-related dysregulation of the HPT axis rather than genuine recovery of thyroid function (6, 13). Moreover, prior studies have shown that normalization of thyroid hormones alone does not necessarily signify endocrine maturity or guarantee improved neurodevelopmental outcomes (4, 6). Likewise, although earlier work has focused primarily on low T4 levels in preterm infants (40), TSH—which more directly reflects anterior pituitary feedback—may act as a sensitive marker of HPT-axis dysregulation in this population (34). Variations in TSH despite normal fT4 levels suggest delayed endocrine maturation rather than true thyroid hormone deficiency, consistent with evidence that preterm infants can exhibit delayed TSH elevation and incomplete postnatal adaptation of thyroid regulation (34). In this context, longitudinal TSH patterns may highlight subtle variations in HPT-axis maturation even when fT4 remains within the normal range, reflecting delayed or incomplete endocrine adaptation rather than overt thyroid hormone deficiency.

Our brain network analysis revealed that elevated TSH levels were associated with altered network centrality in the ACG, SFGmed, and MFG—regions critical for emotional regulation, executive control, and integration of autonomic and cognitive processes. Differences were observed in both BC and DC, indicating early alterations in how key cortical areas communicate and contribute to overall network efficiency.

During the late-gestational to early postnatal window, fronto-limbic circuitry encompassing the ACG, SFGmed, and MFG develops under the guidance of the prolonged subplate scaffold that coordinates early cortico-cortical connectivity (41, 42). Across the first two postnatal years, the association pathways that support these regions (e.g., cingulum, superior longitudinal fasciculus) undergo rapid myelination and reorganization, placing fronto-limbic networks within an extended sensitive period in which deviations in the thyroid axis can delay oligodendrocyte maturation and bias tract development (3, 43–45). Within this neurodevelopmental window, thyroid dysfunction perturbs subplate-related patterning and delays oligodendrocyte maturation, providing a biologically coherent route to atypical brain network development (3, 43). Consistent with fronto-limbic involvement, preterm infants with elevated TSH show diffusion-tractography alterations extending to the middle frontal and middle cingulate gyri with orbitofrontal asymmetry (8); children with CH—despite early treatment—exhibit frontal white-matter and cortical-thickness differences related to early T4/TSH levels (46, 47); and neonatal spectroscopy demonstrates reduced frontal N-acetylaspartate in hypothyroid neonates (48). Together, these data support the interpretation that thyroid-axis disturbances during the subplate-dependent formation and early maturation of association pathways can bias long-range conduction and local synaptic elaboration in fronto-limbic regions, aligning with the centrality differences observed here.

The ACG, a central node within the limbic system, is extensively connected to the amygdala and hypothalamus, mediating emotional reactivity (49, 50) and endocrine responses (51), and the right MFG—a lateral prefrontal node participating in fronto-limbic regulation via interactions with cingulate and amygdala pathways—supports executive and attentional control and top-down modulation of limbic responses (52–54). In our cohort with low-percentile of TSH, concurrent increase in BC and DC in the right ACG, together with reduced DC in the right MFG, indicate reduced path mediation in a limbic hub and fewer effective local connections in a prefrontal limbic-regulatory node, consistent with an early attenuation of limbic–prefrontal integration described in neonatal/preterm connectomics (55, 56). Thyroid-related evidence in preterm cohorts further implicates frontal/cingulate territories, with diffusion-tractography alterations reported in infants with elevated TSH (8).

For clinical practice, our findings favor a two-timepoint interpretation rather than reliance on a single cutoff. Within this framework, infants whose TSH values remained within the lowest quartile at both birth and discharge, or declined from the mid to the lowest quartile across these two time points, demonstrated lower odds of NDI, consistent with more efficient postnatal stabilization of hypothalamic–pituitary–thyroid feedback. In contrast, a birth-screen peak near or above 6 μU/mL with a nondeclining or fluctuating trajectory was associated with higher neurodevelopmental risk, suggesting incomplete adaptation of the HPT axis. Current guidelines recommend levothyroxine treatment for confirmed CH. Persistently higher or increasing TSH levels would ordinarily prompt reassessment for evolving CH and potential initiation of levothyroxine, whereas declining patterns generally indicate spontaneous postnatal adaptation. Based on our present study, persistent higher or increasing TSH levels may reflect delayed maturation of the HPT axis, and although these patterns did not emerge as strong determinants of neurodevelopmental risk in our cohort, they may still hold interpretive value as indicators of regulatory variability that merit continued observation. Accordingly, the trajectory patterns identified here may help differentiate infants with true risk from those showing spontaneous normalization, thereby reducing unnecessary treatment and supporting targeted follow-up. Moreover, DTI-based network metrics at the term-equivalent scan can provide a biologic bridge in trajectory-defined high-risk groups. Building upon these observations, future work should focus on prospective validation using standardized multitimepoint thyroid panels in conjunction with term-equivalent diffusion connectomics, to define clinically actionable thresholds, clarify whether network metrics mediate the association between early thyroid patterns and outcomes, and determine whether trajectory-informed management improves neurodevelopment.

This study has several limitations that should be considered when interpreting the findings. Although infants with severe comorbidities were excluded and IPTW adjustment was applied to reduce baseline differences, these steps cannot fully eliminate residual confounding inherent to prematurity. Major perinatal morbidities, including hypoglycemia and RDS, did not differ between groups, but subtle or cumulative influences on later development remain possible. In addition, detailed maternal thyroid information, including maternal hypothyroidism, hyperthyroidism, or thyroid autoantibodies, was not available. Because maternal thyroid dysfunction can influence fetal thyroid physiology and has been associated with subtle effects on offspring neurodevelopment (57), the absence of these data represents an additional source of potential residual confounding. Accordingly, while unmeasured or incompletely quantified neonatal and maternal factors may still contribute, careful interpretation is warranted, and future studies incorporating broader physiologic and therapeutic variables will be needed to refine these associations.

In summary, longitudinal assessment of thyroid function showed that preterm infants with persistently low or declining TSH trajectories exhibited more favorable developmental outcomes, whereas persistently high or increasing patterns may be associated with postnatal HPT-axis vulnerability and corresponding alterations in frontal–cingulate network organization. These findings suggest that early TSH variation may reflect differences in postnatal HPT-axis maturation relevant to brain network development. Clinically, repeated TSH measurements may improve early identification of infants with delayed endocrine adaptation who may benefit from closer surveillance.

## Data Availability

The original contributions presented in the study are included in the article/**Supplementary Material**. Further inquiries can be directed to the corresponding authors.
