# Supplementary material for: Early postnatal changes in thyroid-stimulating hormone and subsequent neurodevelopment in preterm infants
Source: Front Endocrinol (Lausanne). 2026 Feb 3;16:1745327. doi: 10.3389/fendo.2025.1745327 (PMC12909213; doi:10.3389/fendo.2025.1745327)
Supplement: Supplementary file 1 [file Table1.docx]

**Supplementary Materials**

**Supplementary Table 1. Risk of neurodevelopmental impairment by newborn screening and TEA/discharge TSH quartiles.**

| **TSH quartile** | **Whole-blood TSH range**  **(µIU/mL)** | **Infants, N** | **Neurodevelopmental impairment, N (%)** | **Odds ratio** | ***P*-value** | **Weighted OR (CI)** | **Weighted *P*-value** |
| --- | --- | --- | --- | --- | --- | --- | --- |
| **Newborn screening group** | | | | | | | |
| Lowest quartile | TSH < 2.97 μU/mL | 56 | 23 (41%) | 1.13  (0.59-2.18) | 0.718 | 1.18  (0.57-2.45) | 0.657 |
| Interquartile | 2.97 ≤ TSH < 6.92 μU/mL | 110 | 42 (38%) | Reference | Reference | Reference | Reference |
| Highest quartile | TSH ≥ 6.92 μU/mL | 56 | 30 (53%) | 1.87  (0.97-3.58) | 0.060 | 1.43  (0.72-2.85) | 0.303 |
| **TEA/discharge screening group** | | | | | | | |
| Lowest quartile | TSH < 2.37 μU/mL | 56 | 20 (35%) | 0.68  (0.35-1.32) | 0.256 | 0.65  (0.33-1.28) | 0.211 |
| Interquartile | 2.37 ≤ TSH < 4.76 μU/mL | 109 | 49 (44%) | Reference | Reference | Reference | Reference |
| Highest quartile | TSH ≥ 4.76 μU/mL | 57 | 26 (45%) | 1.03  (0.54-1.95) | 0.935 | 0.95  (0.48-1.87) | 0.879 |

Abbreviations: TEA, term-equivalent age; TSH, thyroid-stimulating hormone; OR, odds ratio; CI, confidence interval.

**Supplementary Table 2.** D**ifferences in global brain network by newborn screening TSH quartile groups.**

| **Global metric** | **Lowest to interquartile (N=166)** | **Highest quartile (N=56)** | ***P*-value** |
| --- | --- | --- | --- |
| Ge | 0.086 ± 0.016 | 0.084 ± 0.016 | 0.355 |
| Le | 0.131 ± 0.026 | 0.129 ± 0.026 | 0.438 |
| Cp | 0.045 ± 0.015 | 0.046 ± 0.015 | 0.805 |
| Lp | 12.115 ± 2.331 | 12.362 ± 2.290 | 0.341 |
| SW | 1.660 ± 0.196 | 1.642 ± 0.195 | 0.445 |

Abbreviations: TSH, thyroid-stimulating hormone; Ge, global efficiency; Le, local efficiency; Cp, clustering coefficient; Lp, shortest path length; SW, small-worldness.
